# NOS2 deficiency has no influence on the radiosensitivity of the hematopoietic system

**DOI:** 10.1186/s13578-018-0228-0

**Published:** 2018-04-27

**Authors:** Chengcheng Li, Yi Luo, Lijian Shao, Aimin Meng, Daohong Zhou

**Affiliations:** 1 Institute of Laboratory Animal Science, Chinese Academy of Medical Sciences (CAMS) and Comparative Medicine Center, Peking Union Medical College (PUMC), Key Laboratory of Human Disease Comparative Medicine, Ministry of Health, Beijing Key Laboratory for Animal Models of Emerging and Remerging Infectious Diseases, Beijing, 100021 China; 2 Department of Pharmaceutical Sciences, University of Arkansas for Medical Sciences, Little Rock, AR USA; 3 Department of Hematology, Tongji Hospital, Tongji Medical College, Huazhong University of Science and Technology, Wuhan, 430030 China; 4 Winthrop P. Rockefeller Cancer Institute, University of Arkansas for Medical Sciences, 4301 W Markham, #607, Little Rock, AR 72205 USA

**Keywords:** Hematopoietic stem cell, Ionizing radiation, NOS2^−^/^−^

## Abstract

**Objective:**

Previous studies have shown that inhibition of inducible NO synthase (NOS2 or iNOS) with an inhibitor can selectively protect several normal tissues against radiation during radiotherapy. However, the role of NOS2 in ionizing radiation (IR)-induced bone marrow (BM) suppression is unknown and thus was investigated in the present study using NOS2^−^/^−^ and wild-type mice 14 days after they were exposed to a sublethal dose of total body irradiation (TBI).

**Methods:**

The effects of different doses of IR (1, 2 and 4 Gy) on the apoptosis and colony-forming ability of bone marrow cells from wild-type (WT) and NOS2^−^/^−^ mice were investigated in vitro. In addition, we exposed NOS2^−^/^−^ mice and WT mice to 6-Gy TBI or sham irradiation. They were euthanized 14 days after TBI for analysis of peripheral blood cell counts and bone marrow cellularity. Colony-forming unit-granulocyte and macrophage, burst-forming unit-erythroid and CFU-granulocyte, erythroid, macrophage in bone marrow cells from the mice were determined to evaluate the function of hematopoietic progenitor cells (HPCs), and the ability of hematopoietic stem cells (HSCs) to self-renew was analysed by the cobblestone area forming cell assay. The cell cycling of HPCs and HSCs were measured by flow cytometry.

**Results:**

Exposure to 2 and 4 Gy IR induced bone marrow cell apoptosis and inhibited the proliferation of HPCs in vitro. However, there was no difference between the cells from WT mice and NOS2^−^/^−^ mice in response to IR exposure in vitro. Exposure of WT mice and NOS2^−^/^−^ mice to 6 Gy TBI decreased the white blood cell, red blood cell, and platelet counts in the peripheral blood and bone marrow mononuclear cells, and reduced the colony-forming ability of HPCs (P < 0.05), damaged the clonogenic function of HSCs. However, these changes were not significantly different in WT and NOS2^−^/^−^ mice.

**Conclusion:**

These data suggest that IR induces BM suppression in a NOS2-independent manner.

## Background

In the 1940s, Jacobson and his colleagues showed that transplantation of splenocytes and bone marrow cells can rescue the life of mice suffering lethal radiation exposure. Lorenz et al. published the similar findings after injecting bone marrow cells into the veins of irradiated mice, which were subsequently protected from radiation damage [1]. In the 1960s, Till and McCulloch discovered hematopoietic stem cells, a discovery that revealed why bone marrow transplantation rescues lethal radiation-induced hematopoietic injury. After transplantation, hematopoietic stem cells can regenerate and differentiate into multipotent progenitor cells in lethally irradiated mice and thus promote the recovery of the hematopoietic system in irradiated mice [2–4].

The hematopoietic system differentiates in a hierarchal manner [5]. Hematopoietic stem cells lie at the top layer of differentiation and are able to self-renew, proliferate and differentiate into different kinds of blood cells. In the normal environment, hematopoietic stem cells are quiescent and maintain a life-long hematopoietic capacity so that the hematopoietic system cannot be exhausted under stress conditions [6]. Multipotent progenitors (MPPs) and hematopoietic progenitor cells (HPCs) are rapidly proliferating cells but have no self-renewal capacity. Following blood loss, hemolysis or infection, MPPs and HPCs can rapidly proliferate and differentiate into mature blood cells. Meanwhile, the function of the hematopoietic system can be recovered.

Nitric oxide (NO) is a toxic, inorganic gas molecule [7]. NO is generated by l-arginine and is catalyzed through three types of nitric oxide synthase (NOS) [8]. According to its origin and function, NOS can be divided into the following three types: neuronal nitric oxide synthase (nNOS or NOS1), inducible nitric oxide synthase (iNOS or NOS2) and endothelial nitric oxide synthase (eNOS or NOS3) [9]. Of these, nNOS and eNOS can together be referred to as constitutive nitric oxide synthase (cNOS), as both are constitutively expressed in vivo and are responsible for the synthesis of basic NO. Further, cNOS regulates various physiological functions, including the information transfer between nerves and cells, as well as vasodilation [10]. However, NOS2 is usually not expressed in resting cells. When cells are stimulated by cytokines, physicochemical factors, or immune microorganisms, the expression of NOS2 can be induced. Meanwhile, a non-physiologic concentration of NO can be synthetized through catalysis, and a series of pathological effects are produced [10].

NOS2 is expressed in multiple cells of the bone marrow, including myeloid cells [11], macrophages [12], megakaryocytes, eosinophilic granulocyte and mononuclear cells [13]. In the bone marrow, eNOS can regulate basic NO, and NOS2 also has a significant influence on the generation and function of basic NO [13]. Under physiological conditions, NO can regulate the proliferation, differentiation and mobilization of hematopoietic stem cells [14]. NO can impact the development and differentiation of hematopoietic cells by regulating the formation of red and granular cell colonies [15]. Vilpo et al. found that NO can reduce the colony-forming ability (CFU-GM) of hematopoietic stem cells and inhibit the growth of T cells and leukemia cells, findings which indicated that nitric oxide donors have potential applications as antineoplastic drugs [16]. High expression of interferon-γ (IFN-γ) or tumor necrosis factor-β (TNF-β) in patients with aplastic anemia can accelerate NOS2 expression in CD34+ cells and may increase the apoptosis rate of CD34+ cells. NO can also affect the expression of certain genes. NO can induce P53 gene mutation, reduce DNA repair capacity and prompt the premature apoptosis of hematopoietic stem cells [17, 18].

Previous studies have shown that ionizing radiation can induce the expression of NOS, including eNOS and NOS2 [19]. In tumor cells, ionizing radiation (IR) can increase the activity of eNOS, promote the generation of intrinsic nitric oxide, and increase murine squamous cell carcinoma (SCCVII) tumor tissue perfusion. Furthermore, IR can cause oxygen enrichment in the tumor tissue and enhance tumor sensitivity to radiotherapy. *Deinococcus radiodurans* (Drad) are resistant to radiation; the killing effect of UV radiation is enhanced in Drad lacking the *nos* gene because of defects in NO synthesis [20]. The above results show that NO can enhance radiation sensitivity to some extent. Furthermore, inhibitors of NOS have a protective effect against exposure [21, 22]. Ultraviolet-B (UVB) can induce NOS2 expression, and a specific inhibitor of NOS2, T1023-a, can relieve superficial injury of blood vessels due to radiation [22]. An inhibitor of eNOS and NOS2, T1023, protects against radiation by decreasing circulatory hypoxia, which is a new and promising radiation protection agent. The primary cause is circulatory hypoxia induced by a reduction in the tissue oxygen supply, which is a result of decreased blood flow volume [21]. Our previous study showed using a gene chip that *Nos2* was upregulated in HSCs 1 month after 6 Gy total body irradiation (TBI), suggesting that NOS2 may play some effect on IR-induced hematopoietic system injury.

In this study, *Nos2* mRNA expression was measured in sorted LSKs by RT-PCR. Bone marrow cells from NOS2^−^/^−^ mice were obtained to observe the influence of IR on the apoptosis and proliferation of bone marrow cells. Additionally, we investigated whether NOS2 deficiency in mice influences bone marrow and peripheral blood cell count decreases or the reduced number and function of HPC and HSC in response to total-body irradiation (TBI) in order to understand the effect of NOS2 deficiency on radiation injury sensitivity.

## Methods

### Mice

C57BL/6J and B6.129P2-*Nos2tm1*Lau/J (NOS2^−/−^) mice were purchased from Jackson Lab (Bar Harbor, MA). Mice were housed at the University of Arkansas for Medical Sciences (UAMS) or St. Jude Children’s Research Hospital, which are both AAALAC-certified animal facilities. Mice received food and water ad libitum. All mice were used at approximately 8–12 weeks of age. The Institutional Animal Care and Use Committees of UAMS or St. Jude Children’s Research Hospital approved all experimental procedures used in this study.

### Irradiation of mice

Mice were exposed to sham irradiation as controls or to a sublethal dose (6 Gy) of TBI in a J.L. Shepherd Model Mark I 137 Cesium γ-irradiator (J.L. Sheperd, Glendale, CA, USA) at a dose rate of 1.14 Gy/min [23]. Dose uniformity was assessed by an independent company (Ashland Specialty Ingredients) with radiographic film and alanine tablets.

### Isolation of blood and bone marrow cells

NOS2^−/−^ and WT mice were subjected to a sublethal TBI dose of 6 Gy. On day 14 post-irradiation, peripheral blood was collected (orbital puncture method). Blood parameters were determined by a Hemavet Instrument (Drew Scientific, Inc.). Bone marrow cells (BMCs) were harvested as described previously [24, 25]. The number of viable BMCs was assessed with trypan blue dye exclusion, and counts were determined with a hemocytometer.

### Colony-forming unit (CFU) assays for BMCs

CFU assays were performed with MethoCult GF (M3434, Stem Cell Technologies) according to the manufacturer’s instructions as described previously [23, 24].

NOS2^−/−^ and WT mice received 6 Gy IR. Bone marrow cells were harvested and placed into 3434 methylcellulose medium 14 days after IR. The CFU for granulocytes and macrophages (CFU-GM) and burst-forming-units for erythroid cells (BFU-E) were measured 7 days later, and the CFUs for granulocytes, erythrocytes, monocytes, and megakaryocytes (GEMM) were measured 12 days later. The colony-forming units were calculated using 10^5^ BMCs. Sections with more than 30 cells were scored as a colony under an inverted microscope according to the manufacturer’s instructions.

### Flow cytometry of HPCs and HSCs after irradiation

Hematopoietic cell phenotypic analysis was completed by flow cytometry. Briefly, 5 × 10^6^ hematopoietic cells harvested from mouse bone marrow were blocked with Fc-block for 10 min, incubated with biotin-conjugated lineage antibodies (CD4, CD8, CD45R/B220, Gr-1, Mac-1 and Ter-119) and then stained with streptavidin-FITC (BD Pharmingen), c-Kit-APC (BD Pharmingen), and Sca1-PE (eBioscience). The frequencies of HPCs (Lin-Sca1-c-kit1 + cells), LSK cells (Lin-Sca1 + c-kit + cells), and HSCs (Lin-Sca1 + c-kit + CD150 + CD48-cells) were analyzed with a flow cytometer (LSRII flow cytometer). Data were analyzed using FlowJo software.

### Isolation of murine Lin^−^ sca-1^+^c-kit^+^ cells (LSK cells)

For the isolation of Lin^–^ cells, BMMNCs were harvested and stained as described above, and the labeled mature lymphoid and myeloid cells were depleted twice by incubation with goat anti-rat IgG paramagnetic beads (Dynal Inc, Lake Success, NY) at a bead-to-cell ratio of approximately 4:1. Cells binding the paramagnetic beads were removed with a magnetic field. The negatively isolated Lin^−^ cells were washed twice with 2% FBS/HBSS and resuspended in complete medium (RPMI-1640 medium supplemented with 10% FBS, 2 mM l-glutamine, 10 μM 4-(2-hydroxyethyl)-1-piperazineethanesulfonic acid (HEPES) buffer, 100 U/ml penicillin, and 100 µg/ml streptomycin; Life Technologies, Grant Island, NY) at 1 × 10^6^ cells/ml. Lin^−^ cells were pre-incubated with anti-CD16/32 antibody (BD Biosciences, San Jose, CA) to block Fcγ receptors and stained with anti-Sca1-PE and c-Kit-APC antibodies, and then LSK cells were sorted with an Aria II cell sorter (BD Biosciences, San Jose, CA).

### Quantitative RT-PCR (qRT-PCR)

Total cellular RNA was extracted from macrophages using the RNeasy Mini kit (QIAGEN, Gaithersburg, MD). Total cellular RNA was extracted from approximately 5000 sorted LSK cells using the Zymo research Quick-RNA Micro Prep kit (The Epigenetics Company, Irvine, CA, USA) according to the manufacturer’s instructions. Reverse transcription was performed immediately using Applied Biosystems’ High Capacity cDNA Reverse Transcription kit (Life Technologies, Grand Island, NY, USA) according to the manufacturer’s instructions. The following *Nos2* primers were used: 5-AGCGCTACAACATCCTGGAGGAAGTGG-3; and 5-GTCCATGATGGTCACATTCTGCTTCTG-3. GAPDH was used as a housekeeping internal reference for mRNA. The Quantitative PCR conditions were as follows: 95 °C for 10 min, 40 × (95  °C for 15 s and 60  °C for 1 min), 95 °C for 15 min, 60 °C for 60 min, and 95 °C for 15 min. All reactions were run in triplicate on an ABI StepOnePlus Real-Time PCR System.

### Cell cycle

Lin- cells were stained with antibodies against various HSC cell-surface markers and were then fixed and permeabilized using Fixation-Permeabilization Solution (BD-Pharmingen, San Diego, CA). Subsequently, cells were stained with anti-Ki67-FITC and 7-aminoactinomycin D (7-AAD) and were then analyzed by flow cytometry.

### Cobblestone area-forming cell (CAFC) assay

The CAFC assay for BMCs was done as described previously [24, 26].

### Statistical analyses

Data were analyzed by analysis of variance (ANOVA) using GraphPad Prism Software (San Diego, CA). In the event that the ANOVA justified post hoc comparisons between group means, the Newman–Keuls or Tukey’s multiple-comparisons test was used. *P *< 0.05 was considered significant. Data are presented as the mean±SEM.

## Results

### *Nos2* mRNA expression is upregulated after 6 Gy total body irradiation

**Fig. 1:**
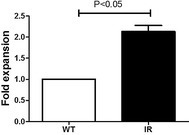
The mRNA expression of NOS2 were decreased in HSCs. The mRNA of NOS2 was detected by RT-PCR. The results are shown as the mean ± S.E

LSKs were sorted 1 month after 6 Gy irradiation. PCR results showed that *Nos2* mRNA was increased in the LSKs of irradiated mice compared with wild-type mice (Fig. 1). The Western blots for verifying NOS2 protein levels were not conducted due to very low numbers of cells available after TBI.

### NOS2 deficiency does not influence hematopoietic cell apoptosis induced by IR

**Fig. 2:**
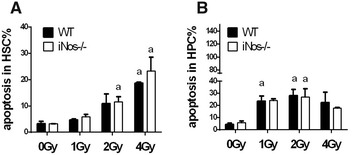
NOS2 knockout does not affect the apoptosis of hematopoietic cells after ionizing radiation. The BM-MNCs of iNOS^−^/^−^ mice and WT mice received different doses of IR (1, 2, and 4 Gy) in vitro and were incubated under 37 °C, and the apoptosis of the BM-MNCs was measured 12 h after IR. **A** The apoptosis rate in HSC. **B** The apoptosis rate in HPCs. The results are presented as the mean ± SE. ^a^P < 0.05 vs. 0 Gy (N = 3)

Mouse BMMNCs became apoptotic 12 h after exposure to ionizing radiation at different doses in vitro. The apoptosis rate among hematopoietic progenitor cells increased with increasing radiation doses (Fig. 2A). However, NOS2^−^/^−^ and wild-type mice showed no significant differences in either HSC or HPC at doses of 0, 1, 2 or 4 Gy (Fig. 2A, B). This result indicates that NOS2 deficiency does not influence hematopoietic cell apoptosis induced by IR.

We next determined the influence of NOS2 on the inhibition of hematopoietic progenitor cell proliferation due to ionizing radiation in vitro. Ionizing radiation can inhibit the proliferation and differentiation of hematopoietic progenitor cells and reduces the function of hematopoietic progenitor cells. The colony forming unit (CFU) assay is an important indicator used to measure the function of hematopoietic progenitor cells.

**Fig. 3:**
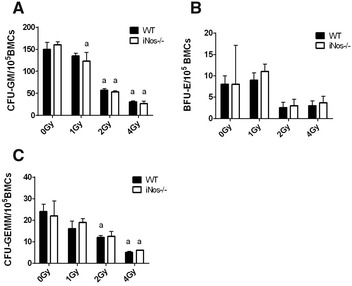
NOS2 knockout did not mitigate the proliferation inhibition of HPCs after IR. The BMCs of NOS2^−^/^−^ mice and WT mice received different doses of IR (1, 2, and 4 Gy) in vitro and were planted into the 3434 methylcellulose medium 2 h after IR. The CFU-GM and BFU-E were measured 7 days later, and the CFU-GEMM was measured 12 days later. The colony forming units were calculated in 105 BMCs **A** CFU-GM in 105 BMCs. **B** BFU-E in 105 BMCs. **C** CFU-GEMM in 105 BMCs. The results are presented as the mean ± SE. ^a^P < 0.05 vs. 0 Gy (N = 5 per group)

The numbers of CFU-GM, BFU-E and CFU-GEMM (P < 0.05) were significantly reduced following 2 and 4 Gy ionizing radiation among mouse bone marrow cells in vitro. The dose of 4 Gy caused more significant damage (*P *< 0.05 compared with 0 Gy); however, after receiving different doses of irradiation (0, 1, 2 and 4 Gy), the CFU-GM, BFU-E and CFU-GEMM of WT and NOS2^−^/^−^ mice in each group were not significantly different (*P *> 0.05). These data indicate that NOS2 deficiency does not affect the apoptosis of hematopoietic cells after ionizing radiation (Fig. 3).

### NOS2 deficiency does not influence radiation-induced decreases in peripheral blood cells

**Fig. 4:**
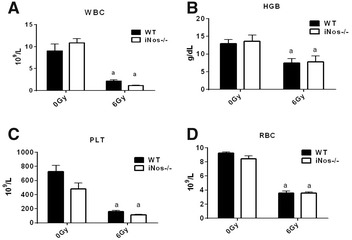
The impact of NOS2 knockout on the number of PBs after 6 Gy IR. NOS2^−^/^−^ mice and WT mice received 6 Gy IR, and the number of PBs were quantified 14 days after IR. **A** The number of WBCs. **B** The number of HGBs. **C** The number of PLTs. **D** The number of RBCs. The results are presented as the mean ± SE. ^a^P < 0.05 vs. 0 Gy (N = 5)

Our previous studies have shown that exposure of C57BL/6J mice to 6 Gy TBI decreases the number of WBCs within 1–2 days after IR. The number of WBCs gradually recoveries from day 14 and almost backs to normal levels by day 30 after TBI. Therefore, in the present study we selected day 14 after IR to examine the role of NOS2 in regulation of the hematopoietic system in response to TBI. The number of mouse peripheral blood was detected 14 days after irradiation. The results are shown in Fig. 4. In both NOS2^−^/^−^ and WT mice, the number of white blood cells (WBC), red blood cells (RBC), hemoglobin (HGB), and platelets (PLT) in the peripheral blood of mice that received 6 Gy irradiation decreased compared to non-irradiated (0 Gy) mice (*P *< 0.05). When comparing NOS2^−^/^−^ and WT mice, the number of peripheral blood was not significantly different after receiving 0 or 6 Gy irradiation (P > 0.05), which indicates that NOS2 deficiency does not influence radiation-induced decreases in peripheral blood cells.

### NOS2 deficiency does not impact the phenotype of bone marrow cells after irradiation

**Fig. 5:**
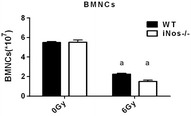
The impact of NOS2 knockout on the number of BMNCs after 6 Gy IR. NOS2^−^/^−^ mice and WT mice received 6 Gy IR, and the number of BMNCs were quantified 14 days after IR. The results are presented as the mean ± SE. ^a^P < 0.05 vs. 0 Gy (N = 5)

**Fig. 6:**
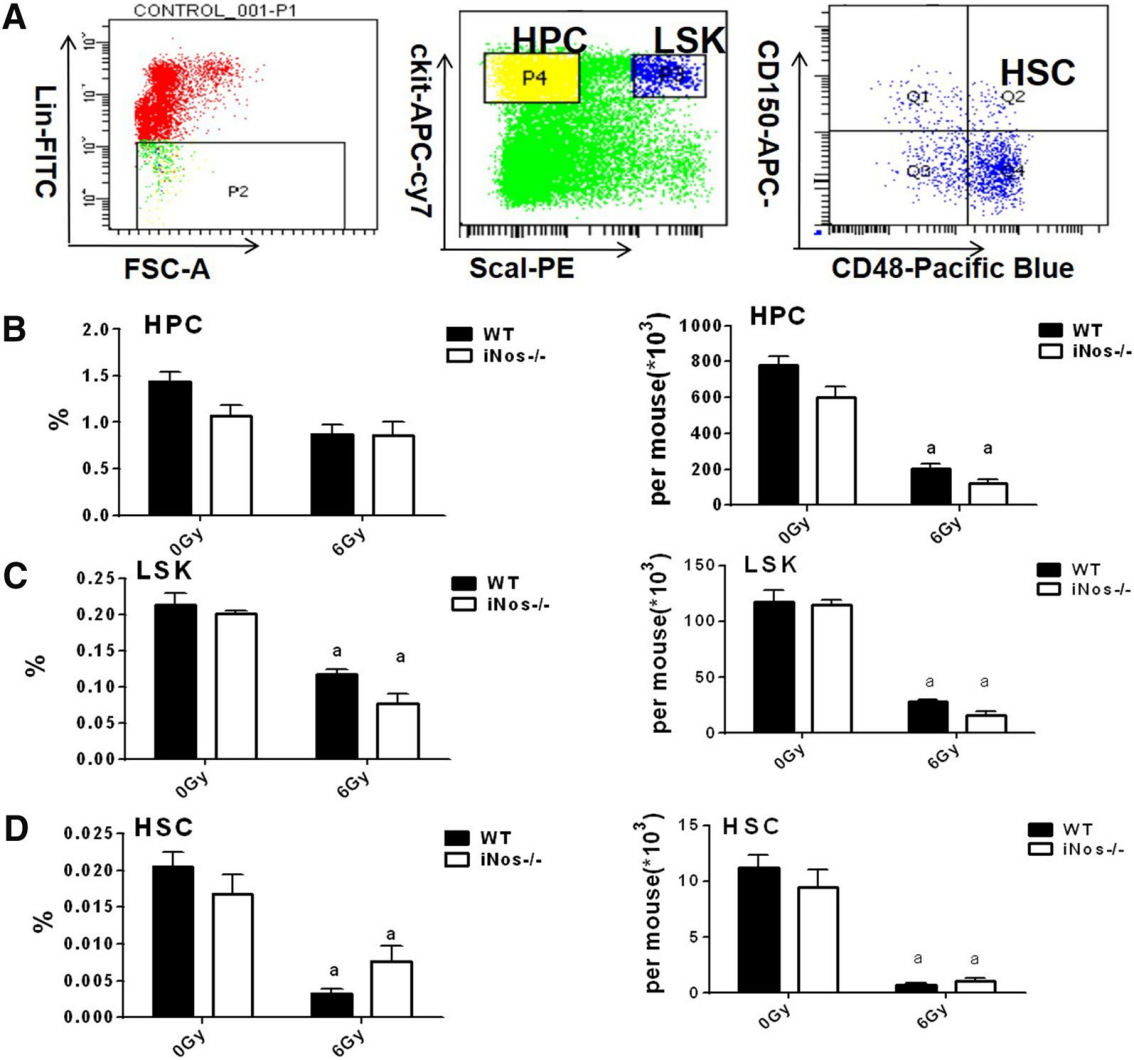
The impact of NOS2 knockout on the phenotype of BMNCs after 6 Gy IR. NOS2^−^/^−^ mice and WT mice received 6 Gy IR, the bone marrow cells were harvest 14 days after IR and analyzed for phenotype by flow cytometry. **A** A representative gating strategy of HPCs (lineage-scal-ckit + cells), LSKs (lineage-scal + ckit + cells) and HSCs (lineage-scal + ckit + CD150 + CD48 + cells) was analyzed by flow cytometry; **B** The frequencies and number of HPCs; **C** The frequencies and number of LSKs; **D** The frequencies and number of HSCs; the data are presented as the mean ± SEM, n = 4

To explore the effect of NOS2 on the radiation sensitivity of the hematopoietic system in mice, the same methods can be adopted as described in “NOS2 deficiency does not influence hematopoietic cell apoptosis induced by IR” section. When comparing NOS2^−^/^−^ and WT mice, the number of bone marrow cells was not significantly different after exposure to 0 or 6 Gy irradiation (*P *> 0.05, Fig. 5). In addition, the phenotype of bone marrow cells in mice was detected by flow cytometry 14 days after irradiation. The gating strategy for flow cytometry analysis are shown in Fig. 6A. The proportion and number of hematopoietic progenitor cells (HPC, Lineage-scal-1-ckit +), LSK cells (Lineage-scal-1 + ckit) and hematopoietic stem cells (HSC, Lineage-scal-1 + ckit + CD48 + CD150 +) were examined. The results are shown in Fig. 6B.

Compared to non-irradiated WT mice, the proportion of HPC, LSK and HSC in WT mice after 6 Gy irradiation declined by 39.56, 44.85 and 85.71%, respectively, while the number of HPC, LSK and HSC decreased by 73.64, 76.00 and 93.06%, respectively (*P *< 0.05). Compared to non-irradiated NOS2^−^/^−^ mice, the proportion of HPC, LSK and HSC in NOS2^−^/^−^ mice after 6 Gy irradiation was reduced by 19.85, 61.38 and 52.94% (*P *< 0.05), respectively, and the number of HPC, LSK and HSC decreased by 79.62, 86.00 and 89.04% (*P *< 0.05), respectively. These results show that 6 Gy TBI in WT or NOS2^−^/^−^ mice significantly changes the phenotype of bone marrow cells after 14 days. The proportion and number of HPC, LSK and HSCs were reduced, which indicates that hematopoietic system injury is caused by TBI. However, compared to WT mice, the percentage and number of HPC, LSK and HSC in NOS2^−^/^−^ mice was not significantly different (*P *> 0.05) after 0 or 6 Gy irradiation exposure. These results indicate that NOS2 deficiency does not affect radiation-mediated decreases in bone marrow cells.

### NOS2 deficiency does not influence IR-induced hematopoietic cell function damage

To determine the effect of NOS2 deficiency on the function of the hematopoietic system, the same methods can be adopted as mentioned in “NOS2 deficiency does not influence hematopoietic cell apoptosis induced by IR” section. Colony forming units (CFUs) and cobblestone area forming colonies (CAFCs) were measured 14 days after irradiation. The results of the CFU assay can mutually corroborate the results of the CAFC at 2 weeks; the results of CAFC at 5 weeks indicate the self-renewal capability of hematopoietic stem cells, which are related to the results of the transplantation experiment.

**Fig. 7:**
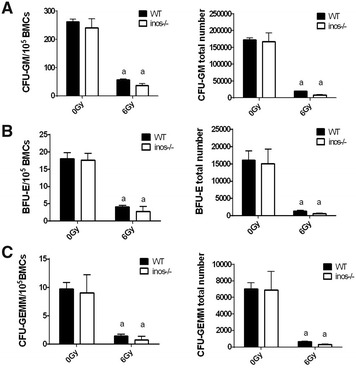
NOS2 knockout did not mitigate the proliferation inhibition of HPC due to ionizing radiation. NOS2^−^/^−^ mice and WT mice received 6 Gy IR. The bone marrow cells were harvested and placed into the 3434 methylcellulose medium 14 days after IR. The CFU-GM and BFU-E were measured 7 days later, and the CFU-GEMM was measured 12 days later. The colony-forming units were calculated in 10^5^ BMCS. **A** The number of CFU-GM. **B** The number of BFU-E. **C** The number of CFU-GEMM. The results are presented as the mean ± SE. ^a^P < 0.05 vs. 0 Gy (n = 4)

The results of the CFU assay show that among WT mice treated with 6 Gy TBI, the CFU-GM number among bone marrow cells was reduced by 78.72% (261.6 ± 10.38 vs. 55.66 ± 4.443, *P *< 0.05) compared to the non-irradiated group (Fig. 7A). The BFU-E number was reduced by 77.69% (17.93 ± 1.904 vs. 4.000 ± 0.577, *P *< 0.05) compared to the non-irradiated group (Fig. 7B). The CFU-GEMM number was reduced by 85.71% (9.666 ± 1.224 vs. 1.388 ± 0.369, P < 0.05) compared to the non-irradiated group (Fig. 7C). Compared to non-irradiated NOS2^−^/^−^ mice, the CFU-GM, BFU-E and CFU-GEMM among NOS2^−^/^−^ mice that received 6 Gy radiation were reduced by 84.76% (240.6 ± 14.43 vs. 36.66 ± 2.651, *P *< 0.05), 84.44% (17.49 ± 0.926 vs. 2.722 ± 0.611, *P *< 0.05) and 91.97% (9.000 ± 1.452 vs. 0.7222 ± 0.264, *P *< 0.05). Our results show that ionizing radiation at a dose of 6 Gy can significantly reduce the proliferation of hematopoietic progenitor cells. However, when WT mice are compared to NOS2^−^/^−^ mice, hematopoietic cell apoptosis and function are not significantly different at 0 or 6 Gy irradiation, which indicates that NOS2 deficiency has no influence IR-induced hemopoietic progenitor cell damage.

**Fig. 8:**
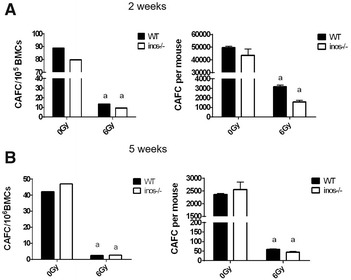
NOS2 knockout did not influence the IR-induced HSC function decrease. NOS2^−^/^−^ mice and WT mice exposure to 6 Gy irradiation. The bone marrow cells were harvested and planted on the FBMD1 stromal cells. The clonogenic function of HPCs and HSCs in BM-MNCs was measured after 2 and 5 weeks, respectively. The CAFCs were calculated in 10^5^ BMCS. **A** The number of CAFCs at week 2. **B** The number of CAFCs at week 5; the results are presented as the mean ± SE. ^a^P < 0.05 vs. 0 Gy (n = 4)

The cobblestone area forming cell (CAFC) assay can evaluate the function of hematopoietic stem cells and hematopoietic progenitor cells in vitro. Bone marrow cells from WT mice and NOS2^−^/^−^ mice were exposed to 0 or 6 Gy TBI and were then plated on FBMD1 stromal cells. CAFCs were detected after 2 and 5 weeks to measure the function of hematopoietic progenitor cells and hematopoietic stem cells. The results of CAFC for 2 and 5 weeks show that 6 Gy TBI reduced the function of hematopoietic stem cells and hematopoietic progenitor cells in WT and NOS2^−^/^−^ mice. However, there was no difference between the WT and NOS2^−^/^−^ mice after receiving 0 or 6 Gy irradiation (Fig. 8A, B), which shows that NOS2 deficiency does not influence hematopoietic stem cell function damage after irradiation.

### NOS2 deficiency does not impact IR-induced cell cycle arrest

**Fig. 9:**
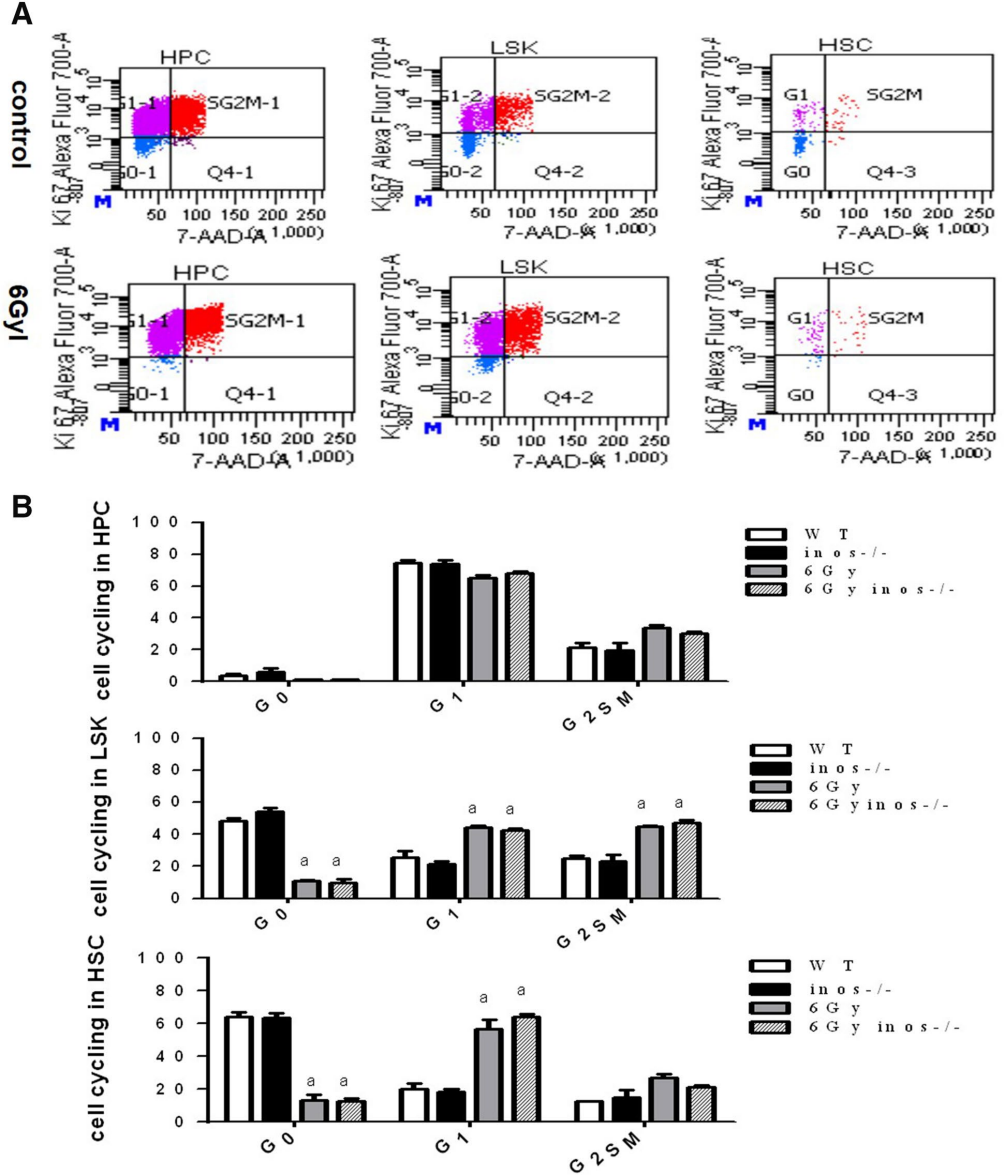
NOS2 knockout has no influence in the cell cycling arrest of BMNCs after 6 Gy ionizing radiation. NOS2^−^/^−^ mice and WT mice received 6 Gy IR. and analyzed for cell cycling by flow cytometry. **A** A representative gating strategy of cell cycling was analyzed by flow cytometry; **B** The analysis of cell cycling in HPCs, LSKs and HSCs. The data are presented as the mean ± SEM (n = 4)

To observe the effect of NOS2 deficiency on the homeostasis of hematopoietic cells, WT and NOS2^−^/^−^ mice were exposed to 0 or 6 Gy TBI. The cell cycle status of hematopoietic cells was then detected. For irradiated WT and NOS2^−^/^−^ mice, the percentages of cells in the G0 period were as follows: HSC, 64.1 and 63.6%, respectively; LSK, 46.1 and 56.6%, respectively; and HPC, 3.8 and 5.9%, respectively (Fig. 9B). These data show that a small number of cells at a higher degree of differentiation will be in G0. Conversely, the proportion of cells in S–G2–M phase will be larger. The percentage of HPC, MPP and HSCs in G0 was lower after 6 Gy TBI. Meanwhile, the proportion in S–G2–M was significantly increased, which indicates that TBI induces hematopoietic cells to cycle. However, when comparing WT and NOS2^−^/^−^ mice, there was no significant difference after either 0 or 6 Gy IR. Thus, NOS2 deficiency does not influence the cell cycle change that is induced by TBI.

## Discussion

Exposure to IR leads to increased oxidative stress, DNA damage, genomic instability and increased inflammation; NOS2 is implicated in regulation of these processes [27–30], but its role in radiation responses has not been investigated. Gene chip results showed that *Nos2* is upregulated in HSCs 1 month after 6 Gy TBI (data not shown), and this result has been further verified by RT-PCR using sorted LSKs. The present study examined the role of NOS2 in response to IR using a NOS2-deficient mouse model.

Our results showed that 1, 2, and 4 Gy irradiation induces defects in apoptosis and colony forming ability among hematopoietic progenitor cells in NOS2^−^/^−^ and WT mice in vitro. There were no significant differences between NOS2^−^/^−^ and the WT group, suggesting that the NOS2 deficiency does not affect the sensitivity of hematopoietic cells in vitro. Michurina et al. found that NOS inhibitors do not affect the apoptosis and survival of hematopoietic stem cells in vitro [13]. Our results showed that the apoptosis and CFU of bone marrow cells in NOS2^−^/^−^ mice showed no significant difference compared with that of WT mice after exposure to 1, 2, and 4 Gy TBI. These results demonstrated that physiological NO does not affect the function of hematopoietic cells in vitro. One reason may be because a relatively small amount of NO production is induced in hematopoietic cells by NOS2 under the physiological state [13]. Early studies showed that low concentrations of NO donor decreased the CFU ability of CD34+ cells in human bone marrow [15], suggesting that exogenous NO can damage the differentiation of hematopoietic cells.

To explore the effect of NOS2 on the hematopoietic system, WT and NOS2^−^/^−^ mice received sham irradiation (0 Gy) and 6 Gy TBI; the results showed that 6 Gy TBI leads to decreased numbers of peripheral blood cells and bone marrow cells, damages the function of HSPCs, and breaks hematopoietic stem cell homeostasis in WT mice. HSCs under resting state are important for resisting harmful stimuli, maintaining stemness, and preventing HSCs from premature exhaustion [31]. The NOS2^−^/^−^ mice showed the same results after 6 Gy TBI. Previous studies showed that NO induce cell cycle arrest by regulating the cell cycle protein dependent kinase inhibitor p21WAF1 [32]. To inhibit NOS activity, we can increase the number and self-renewal capacity of hematopoietic stem cells [13]. Our results showed that 6 Gy TBI induces bone marrow cell entry into the cell cycle in NOS2^−^/^−^ mice; HSC went from the G0 phase to G2SM phase, while there was no significant difference between WT mice and NOS2^−^/^−^ mice.

The present study showed that induction of HSC damage by IR is independent of NOS2, because NOS2 deficiency in mice had no significant effects on IR-induced bone marrow cell number and colony forming ability. Furthermore, the lack of an effect of NOS2 deficiency cannot be attributed to the cell cycling arrest induced by IR. These data suggest IR induces BM suppression in a NOS2-independent manner, even though NOS2 is upregulated in HSCs after 6 Gy TBI.

Previous studies have shown that LPS, TNFα, IL-17 and other modulators can induce the activation of NFκB and MAPK in bone marrow; therefore, NOS2 expression can be stimulated, sufficient NO can be produced, and the function of bone marrow cells can be reduced. High expression IFN-γ of TNF-β in the bone marrow cells of aplastic anemia patients can accelerate NOS2 expression of CD34+ cells and result in the apparent increase in CD34+ cell apoptosis rate. However, IR-induced NOS2 activation in bone marrow cells is seldom researched; a previous study showed that the specific inhibitor of NOS2, T1023-a, can relieve superficial injury of blood vessels due to radiation [33], which has a protective effect against radiation by decreasing circulatory hypoxia. In our study, NOS2 expression was inhibited by NOS2 deficiency, which did not rescue IR-induced hematopoietic system damage.

Reactive oxygen species (ROS) induced by IR could damage HSCs by interfering with p38MAPK [34]. Even though T lymphocyte dysfunction has been reported to be attributable to nitrative stress induced by reactive nitrogen species (RNS) [35], there is still no evidence to demonstrate that HPCs and HSCs can be damaged by IR-induced RNS. A previous study showed that LPS induces oxidative DNA damage in the bone marrow of mice in an NOS2-dependent pathway [36], suggesting that the oxidative stress induced by IR may be different from that induced by inflammation.

However, whether the reactive nitrogen species (RNS) induced by NOS2 after ionizing radiation in the hematopoietic system shows pathological injury remains to be investigated [37]. LPS, TNFα and IL-17 induce the expression of NOS2 by activating NFκB and MAPK [38, 39]; NO could damage bone marrow function, suggesting that the oxidative stress induced by IR is different from that induced by inflammation. It is possible that the increased production of reactive active oxygen (ROS) and activation of p38MAPK as reported previously [34, 40]. In addition, activation of NFkB was also reported to be involved in IR-induced bone marrow suppression [41]. Furthermore, HSCs from p53-deficient mice are less sensitive to IR than are those from wild-type mice and treatment with a p53 inhibitor protected mice from IR induced lethal damage by suppression of p53-dependent apoptosis [42]. Finally, activation of the G-CSF/Stat3/BATF pathway can also contribute to IR-induced HSC injury via promoting HSC differentiation [5].

In addition, IR damages the hematopoietic system function through multiple pathways as follows: IR can induce DNA damage, resulting in cell apoptosis; IR can inhibit hematopoietic stem cell differentiation; and IR can activate oxidative stress in stem or progenitor cells and promote senescence [5, 43]. As a consequence, the damaging effect of these pathways may compensate for the cytotoxic effects caused by NOS2 deficiency.

Studies in knockout mouse models of p53, targets of p53, CCAAT/Enhancer-Binding Protein Delta (C/ebpd) and other genes led to the identification of some of the molecular mechanisms that drive cellular, tissue, and organismal responses to radiation [23, 44, 45]. However, additional mechanisms that contribute to the radiation response remain undefined, and investigations in this direction will aid in the development of novel, target-specific interventions to protect normal tissues from radiation injury [46]. Christopher et al. reported that NOS2a acts downstream of the transcription factor C/ebpb to control expansion of HSPCs following infection [47]. Our study suggested that manipulating NOS2 activity does not influence the capacity of mammalian HSPCs. In the future, a model with hematopoietic system-specific NOS2 deficiency may help to exclude the compensation effect of other NOS enzymes. The present study showed that IR induced BM suppression in a NOS2-independent manner at 14 days after TBI, the delayed changes need further investigation.

## Conclusion

The present study showed that IR induces BM suppression in a NOS2-independent manner, even though NOS2 is upregulated in HSCs after 6 Gy TBI.

## Abbreviations

BM: bone marrow
BMMNCs: bone marrow mononuclear cells
BFU-E: burst-forming unit erythroid
CFU-GM: colony forming unit-granulocyte, macrophage
CFU-GEMM: colony forming unit-granulocyte, erythroid, macrophage, megakaryocyte
CAFC: cobblestone area-forming cell assay
cNOS: constitutive nitric oxide synthase
DNA: deoxyribonucleic acid
DPI: diphenylene iodonium
DMSO: dimethyl sulfoxide
eNOS: endothelial nitric oxide synthase
FBS: fetal bovine serum
FITC: fluorescein isothiocyanate
FBMD-1: flask bone marrow dexter-1
HGB: hemoglobin
HPCs: hematopoietic progenitor cells
HSCs: hematopoietic stem cells
IR: ionizing radiation
NOS2: inducible nitric oxide synthase
IFN-γ: interferon-gamma
Lin-BMCs: lineage-negative bone marrow cells
LYM: lymphocyte
LPS: lipopolysaccharide
MFI: mean fluorescence intensity
MPP: multipotent progenitor
MAPK: mitogen activated protein kinases
NF-κB: nuclear factor-kappa beta
NOS: nitric oxide synthase
nNOS: neuronal nitric oxide synthase
p38MAPK: p38 mitogen activated protein kinases
PI: propidium iodide
PLT: platelet
ROS: reactive oxygen species
RT-PCR: reverse transcriptase-polymerase chain reaction
qRT-PCR: quantitative RT-PCR
RBC: red blood cell
TBI: total body irradiation
TNF-β: tumor necrosis factor-beta
WT: wild-type
WBC: white blood cell
